# Five Cases of Bone Packing-Senn’s Method

**Published:** 1891-03

**Authors:** P. O’Keef

**Affiliations:** Oconto, Wisconsin


### Southern Medical Record.

A MONHLY JOURNAL OF MEDICINE AND SURGERY.

VOL. XXI. ATLANTA, GA., MARCH, 1891. No. 3.

### Original Articles.

### FIVE CASES OF BONE PACKING—SENN'S METHOD

BY P. O'KEEF, M. D., OCONTO, WISCONSIN.*

CASE 1.—L. L., male, aged 4 years, was brought to my office by Dr. Paramore on July 17, 1890. I found a small opening discharging pus above the right external malleolus. An incision two inches long exposed the necrosed lower extremity of the fibula, which was grasped by sequestrum forceps and the entire shaft of the bone removed as it was completely detached. After careful irrigation with a 1-1000 sublimate solution, I pushed decalcified bone chips up from the lower end of the canal until the cavity was filled moderately firm. The wound was now sutured, and an antiseptic dressing applied. On removing the dressing, ten days later, the wound was healed. Patient began to walk in three and a half months and is now perfectly well.

CASE 2.—J. H., aged 8 years, male, came to me July 29th, 1890. There was a suppurating sore above internal malleolus of the right leg; on pressure below the knee pus flowed freely from the opening. The tibia was found to be necrosed as far as the probe would reach; I therefore made an incision along the crest of the tibia from below upward to the tubercle. The periosteum seemed to be completely detached, but on using

*Read before the Brainard Medical Society.

the chisel a narrow strip of bone from one–quarter to one-half inch wide (the internal border of the bone) retained its periosteal attachment and was carefully preserved. With this exception the entire shaft and the cancellous structure of the head of the tibia was completely removed.

The cavity was thoroughly irrigated and packed with decalcified bone chips, healing being complete in fourteen days under two dressings. The parents now called my attention to a swelling below the outer extremity of the left clavicle and to an enlargement of the second phalanx of the right middle finger. I removed the shaft of the phalanx and packed with bone chips. I then proceeded to remove the tuberculous patch below the clavicle by removing the whole thickness of the skin, with this a small spicula of bone came away and the outer end of the clavicle had to be removed. This was done subperiosteally and treated as the other bones were. Patient made a good recovery and is now able to walk about.

CASE 3.—M. W., female, aged 13 years, came to my office August 31, 1890. She said she was out picking berries last week and sprained her ankle. Her right leg was swollen, red and very painful, fluctuation distinct over lower half. An incision was made from above the ankle to within two inches of the tubercle of the tibia. Below this the periosteum was entirely detached and the tibia surrounded with pus. The shaft of bone from this point to the ankle was entirely removed and the cancellous structure of the lower extremity gouged out. The cavity was irrigated and packed with bone chips, periosteum and skin sutured with catgut and silk and an antiseptic dressing and binder's board splint applied. Two days later I called to see the patient and found the dressings scattered about the floor. After washing the leg in a 1-1000 sublimate solution, I applied new dressings. I removed the dressings a week later and found a little pus at the site of drainage. This I washed away with a 1-1000 sublimate solution, and some of the chips also came away. The leg was now dressed every day for two weeks, when the remaining bone chips were entirely buried in granulations and patient went home. I saw her about a month later, the wound was entirely healed, but there is a depression at the lower end of bone where the chips came away. The result is better, however, than I expected two days after the operation. Patient has not been allowed to walk yet.

CASE 4.—J. McD., aged 6 years. Operation August 18, 1890. The entire shaft and head of the left tibia, except the articular lamella, were removed and the cancellous tissue of the lower extremity of the bones cooped out. After irrigating the cavity I was about to pack it with bone chips, when, on putting my hand under the leg to place it in proper position, pus welled into the cavity through an opening in the periosteum at its upper extremity. On examination with the finger the intermuscular spaces in calf were found to be filled with pus. These pockets were thoroughly curetted and irrigated with a large quantity of a 1-1000 sublimate solution. The leg was now held in position and the periosteum filled with decalcified bone chips, the wound sutured and a plaster-of-Paris bandage applied from the hip to the foot over the antiseptic dressing. Wound entirely healed in ten days under first dressing. The bone is now firm, boy able to walk about, and the only deformity is a slight projection outwards of the head of the fibula. In this case, like Case 3, the patient never complained of any pain until four or five days before the operation, then while playing out of doors the leg suddenly gave way under him. At the time of the operation the head and shaft of the bone were separated from each other.

CASE 5.—Exsection of hip joint. Mary T., aged 9 years, operation May 4, 1890. After removing the head and the neck of the femur, the cartilage of acetabulum was found to be eroded and this cavity was thoroughly scraped with the sharp spoon. As the patient lived eight miles from my office, I thought that if I could close the wound at once and get healing by first intention instead of the established practice and leaving the wound open and packed with gauze until the cavity filled by granulation, I would avoid a great deal of annoyance to my patient and myself many rides.

After curetting the capsule under irrigation with a 1-1000 sublimate solution, I filled the cavity with bone chips and closed the wound with a sufficient number of buried catgut sutures to bring the surfaces into apposition, the skin was sutured with silk, drainage being secured by catgut. Immobilization was secured by a plaster-of-Paris bandage from the knee to the umbilicus over the dressings, a large sequestrum being left through which the wound could be examined and dressed. Healing was complete in fifteen days under two dressings. Patient is able to walk about. Anchylosis is almost complete, there is half an inch shortening, but the hip is fully as prominent as its fellow.

There was never any temperature after the operation, and the pain, which before the operation was not entirely relieved by four to five grains daily of morphia, only required one-eighth grain dose after the operation.

